# Proceedings of the Society

**Published:** 1856-04

**Authors:** 


### QUARTERLY REPORT OF THE PROCEEDINGS OF THE EPIDEMIOLOGICAL SOCIETY.

### PAPERS READ AT ORDINARY MEETINGS.

*Monday, January* 7, 1856. "On the Prophylaxis of Cholera by some of the Vegetable and Mineral Acids." By J. H. Tucker, Esq.

*Monday, February* 4. "Observations on the Cholera Epidemic that occurred in 1854 at St. Laurent d'Aigouze and its environs." By Mons. Raymond Falot. Read by Dr. Camps.

*Monday, March* 3. "On the Pathology of Cholera." By Philip B. Ayres, M.D.

At this meeting the nomination of office-bearers for the ensuing year took place.

### REPORTS.

The Report of the Epizootic Committee on "Pleuro-pneumonia among Cattle," drawn up from the replies to the queries issued some time back, has been received from the secretary of the Committee, and will be read at an early meeting of the Society.

### NEW MEMBERS.

*Resident.* William Odling, M.B., Professor of Practical Chemistry and Natural Philosophy in Guy's Hospital; Robert Nichol, M.D., Hampstead.

### CORRESPONDING MEMBERS.

George Lee, M.D., Rio de Janeiro; Robert Lallemant, M.D., Rio de Janeiro; E. H. Barton, M.D., New Orleans.

